# Comment on “The translational challenge in Chagas disease drug development” by Kratz et al.

**DOI:** 10.1590/0074-02760210501chgsb

**Published:** 2022-05-23

**Authors:** Manu De Rycker

**Affiliations:** 1 University of Dundee

In their comprehensive review Kratz et al. lay out the many challenges faced by the Chagas disease (CD) drug discovery community. The lack of new compounds entering clinical trials is to a large extent due to these challenges, as well as the relatively limited amount of funding available for this neglected tropical disease. While the pipeline remains sparse, our understanding of how to develop a new CD drug has improved significantly since the clinical trial failures of the CYP51 inhibitors posaconazole and fosravuconazole.

An important point made in this review is that most drug discovery groups are aiming to achieve sterile cure in an animal model before progressing compounds to the clinic. This is a very high barrier, and one that is not required for most infectious diseases. However, the long-term persistence of small numbers of parasites during the indeterminate phase of CD and the ensuing pathogenesis indicate that such compounds will have the highest likelihood of clinical success. The intrinsic ability of a compound to achieve sterile cure relies on its mode-of-action. In our experience, few modes-of-action are able to kill all parasites *in vitro*, even after long treatments with high compound concentrations. We refer to the survivors as persisters, and fundamental biology studies are ongoing to better understand their nature in several groups. To predict the ability of compounds (and thus modes-of-action) to achieve *in vivo* sterile cure, we profile all series of interest in *in vitro* washout outgrowth assays, where long treatments of intracellular parasites (up to 16 days) are followed by an outgrowth period of two months. If no parasites are detected during these two months, we consider this *in vitro* sterile cure. Based on the data obtained with benznidazole and posaconazole, the ability to achieve “cellular cure” in this model is a prerequisite for full efficacy in a mouse model of chronic CD (i.e., no relapse after three rounds of immunosuppression and *ex vivo* imaging). In addition to the right mode-of-action, compounds also need to exhibit appropriate pharmacokinetics. Much knowledge remains to be gained in this area. As pointed out in the review, *Trypanosoma cruzi* infection dynamics in animals are complex, and involve (temporary) infection of many different tissues. The challenge for drug discovery is to develop compounds that reach all parasite reservoirs and maintain sufficiently high concentrations for long enough to kill all parasites. To understand the required distribution properties better, further investigation of tissue distribution for compounds that achieve sterile cure is necessary. Importantly, the goal is not to develop drugs for mice but for humans. Understanding of how parasite dynamics and distribution translate to the human patient situation is key, but not easily achieved. The most pragmatic option going forward is to take the best compounds, based on *in vitro* data and animal studies, to human clinical trials. There is also value in back-translating clinical outcomes, which is somewhat confounded by the reactive nature of benznidazole and nifurtimox and therefore potential disconnection between pharmacokinetics and pharmacodynamics, but which has nevertheless brought key insights into the causes for the clinical failures of the CYP51 inhibitors. These insights have radically changed the drug discovery pathway for CD drug discovery.

As pointed out by the authors, target-based work has been relatively unsuccessful for CD, due to the lack of validated targets as well as poor translation between genetic and chemical validation. Many proteins are essential to the parasite, however this does not guarantee that chemical interference with these targets using small molecules will have a fast killing effect on all parasite forms, including slow replicating ones. One potential route to circumvent this problem is through the development of PROTAC drugs. These compounds mimic genetic knockouts by degrading the target and may improve the success rate of target-based programmes. Further fundamental science is imperative to understand these parasites better, and in particular to identify targets that are essential across all relevant life-stages. Phenotypic programmes themselves often reveal new and interesting targets and several groups have found that target-deconvolution from phenotypic programmes is extremely valuable as it allows to build enzyme/binding assays, can enable structure-guided drug discovery and increases understanding and ability to mitigate toxicity risks.

The authors briefly touch on the option of combination therapy. Combination therapy has been applied successfully in many other infectious diseases and in view of the likely need for sterile cure this approach is indeed worthwhile exploring for CD. The “double-hit” resulting from targeting two appropriate modes-of-action in the parasite may be an effective way to kill all forms of the parasite. While monotherapy remains the primary goal, we believe the community should further explore combination strategies that have the potential to give a fast and effective cure. The authors also touch on the “awakening” strategy, aiming to increase replication in persister/dormant parasites, and while speculative and possibly even risky, this approach is being pursued in other infectious diseases where persisters pose a problem and is worthwhile investigating.

Patients and other stakeholders should not be disheartened by the many challenges posed by this parasite. The Chagas drug discovery community may be relatively small but has made significant progress in understanding the path to the clinic for new compounds. Only by progressing new compounds to the clinic we will understand the true translational value of the new paradigms developed since the failure of the CYP51 inhibitor trials. Ongoing concerted and scientific approaches will eventually address the remaining key questions, provided funders across the world keep investing in this area, both at the fundamental science level and in drug discovery. I fully agree with the authors that a spirit of openness and collaboration is essential in this area to maximise the benefit of any new understanding and accelerate CD drug discovery.
